# Corporate social responsibility to improve access to medicines: the case of Brazil

**DOI:** 10.1186/s12992-017-0235-7

**Published:** 2017-02-21

**Authors:** Halla Thorsteinsdóttir, Natasha Ovtcharenko, Jillian Clare Kohler

**Affiliations:** 1 Institute of Health Policy Mangement and Evaluation, Dalla Lana School of Public Health, University of Toronto, Toronto, Canada; 2 Small Globe, Toronto, Canada; 3 School of Medicine, Queens University, Kingston, Canada; 4 Leslie Dan Faculty of Pharmacy, University of Toronto, Toronto, Canada; 5 Munk School of Global Affairs, University of Toronto, Toronto, Canada; 6 Dalla Lana School of Public Health, University of Toronto, Toronto, Canada

**Keywords:** Access to Medicine, Brazil, Corporate Social Responsibility, Health policies, Industry-Government alignment, Pharmaceutical industrialization, Universal health care system

## Abstract

**Background:**

Access to medicines and the development of a strong national pharmaceutical industry are two longstanding pillars of health policy in Brazil. This is reflected in a clear emphasis by Brazil’s Federal Government on improving access to medicine in national health plans and industrial policies aimed at promoting domestic pharmaceutical development. This research proposes that such policies may act as incentives for companies to pursue a strategic Corporate Social Responsibility (CSR) agenda. CSR that supports Governmental priorities could help companies to benefit significantly from the Governmental industrial policy. We sought to determine whether CSR activities of Brazilian pharmaceutical firms are currently aligned with the Federal Government’s health prioritization. To do so we examined key Brazilian health related policies since 2004, including the specific priorities of Brazil’s 2012–2015 Health Plan, and compared these with CSR initiatives that are reported on the websites of select pharmaceutical firms in Brazil.

**Results:**

Brazil’s national health plans and industrial policies demonstrated that the Federal Government has followed diverse approaches for improving access to medicines, including strengthening health care infrastructure, increasing transparency, and supporting product development partnerships. Case studies of six pharmaceutical firms, representing both public and private companies of varying size, support the perspective that CSR is a priority for firms. However, while many programs target issues such as health infrastructure, health care training, and drug donation, more programs focus on areas other than health and do not seem to be connected to Governmental prioritization.

**Conclusion:**

This research suggests that there are loose connections between Governmental priorities and pharmaceutical firm CSR. However, there remains a significant opportunity for greater alignment, which could improve access to medicines in the country and foster a stronger relationship between the Government and industry.

## Background

The Federal Government of Brazil has had a longstanding and ambitious policy to increase access to medicine for its population through pursuing the twin strategies of establishing a universal health care system and by promoting domestic pharmaceutical industrialization [1–3]. This approach reflects how the Government aims to improve access to medicines for its population and at the same time promote economic development through further industrialization. In the past decade, the Government’s priorities in this area have shifted from a focus on the local generic industry to promoting research and development (R&D) capabilities in the private sector. This further strengthens the ability of the domestic pharmaceutical industry to improve the supply of pharmaceutical products and support the Government’s universal health care program. This shift and continued support of the domestic industry has been accompanied by efforts of the domestic industry to take a more active role in the country’s health sector through corporate social responsibility (CSR) policies.

The significant expertise and costs associated with the development and testing of new drugs are such that the process is typically only within the reach of large private corporations. Even though universities, and other public sector organizations, are heavily involved in basic research in the pharmaceutical area, the development of promising drug candidates is, in most cases, licensed to private companies. To engage in R&D in this sector requires a large amount of resources, which in the case of Brazil are provided by the Federal Government, largely through research funding and by promoting public-private partnerships. The Government has, for instance, invested in vaccine research by state-owned laboratories such as Fiocruz and the Butantan Institute and supports public-private partnerships such as that between Fiocruz and GlaxoSmithKline [1].

There can be various drivers for pharmaceutical companies to take part in CSR [4]. Clearly, CSR helps promote a positive image of a company and is particularly helpful if a company has had a sullied reputation in the past. What is more, initiatives such as the Access to Medicines Index (ATM), funded by the Gates Foundation, is gaining notice given that it ranks how well select companies are doing from a societal standpoint. The ATM thus aims to put positive pressure on companies to engage in activities that fit this agenda, as companies are being compared against each other. Major international efforts to track and assess companies based on CSR offer the potential for strong programming or lack thereof to become associated with public approval. Efforts by companies to improve their standing on measures such as the ATM shows that they strive to improve their practices to reflect their commitment to CSR and also that they understand that good CSR can help improve their bottom line - more engaged employees and possibly shareholders that are more willing to invest in a company that is perceived as doing good works [4].

The Brazilian Federal Government has used two key strategies in attempts to achieve its goals for the health system: improving the governance of the health system to be more responsive to citizen needs and supporting domestic production and innovation of pharmaceuticals to decrease dependency on foreign import [5]. As the Government supports its domestic industry, there is an incentive for companies to strengthen their own CSR practices to reflect the Government’s two key strategies. Such action would create a cooperative relationship between the Federal Government and the private sector, as they support one another to attain the same public health goals.

This paper examines the Brazilian Federal Government’s strategies to improve access to medicine by exploring its key health related prioritizations. It also examines the integration and alignment of the domestic pharmaceutical industry with Government prioritization through an analysis of CSR initiatives of Brazilian firms. Specifically, this study analyzes the health policies of the Workers Party Presidents, Luiz Inacio Lula da Silva (2002–2010) and Dilma Rousseff (2011–2016), and how their health policies promoted access to medicines and innovation. We assess the key goals of the 2004–2007 National Health Plan, the 2006 Health Pact, the 2007 Mais Saúde Plan, the 2009 Brasil Maior Plan, and the 2012–2015 Health Plan. We further rely on information on pharmaceutical companies’ CSR activities, provided by them on their websites and in reports, to evaluate the alignment of the Brazilian pharmaceutical industry with the Federal Government’s areas of prioritization.

This research is highly relevant for various reasons, including the growth of Brazil’s pharmaceutical market, the importance Brazil’s Federal Government puts on health and access to medicines, and the value that the Government puts into fostering the domestic pharmaceutical industry. Some degree of reciprocity between the Government and the domestic industry could yield positive results for Brazilian citizens. Further, in light of the corruption scandals that recently led to an impeachment and suspension from the Brazilian Government of President Dilma Rousseff, it seems likely that citizens will be even more sensitized to the importance of the investment and operation policies and practices in the country to ensure that they are geared towards the public good.

The paper is organized as follows. First, the literature review will be presented on the concepts of CSR, followed by a review of literature on access to medicine and CSR in Brazil. We will then address the key research questions of this paper and analyze Brazil’s health related policies as well as the CSR areas focused on by Brazilian pharmaceutical firms, and examine their alignments. Lastly, we will present the main messages drawn from this research.

### CSR and the private sector

The global pharmaceutical market is enormously lucrative. Annual global pharmaceutical spending is forecasted to reach US$1.2 trillion by 2016 and annual spending growth will increase from US$30 billion in 2012 to US$70 billion in 2016 [6]. Pharmaceutical expenditures are typically one of the top healthcare expenditures for governments globally and can reach as high as 50% of the total health spending in some developing countries [7]. Brazil is no exception, as its pharmaceutical market is the second largest in the emerging world, with the value of the prescription drugs sold to Brazil’s middle class in 2010 valuing at $8 billion dollars [8].

Such high levels of profitability have been increasingly exposed to new standards and regulatory frameworks, as a result of increased pressure from employees, community groups, non-governmental organization (NGOs) and governments to engage in activities that are morally and ethically sensitive [9]. This phenomenon, called CSR, is defined as when companies actively do “good” outside of the scope of their business, often with a complementary strategic goal of securing the loyalty of customers [4]. While the reason behind the emergence of CSR is based on ‘doing the right thing’ without taking into consideration the financial implications of such behaviour, companies are also aware that CRS has a strategic component, as it allows for companies to add value to their products by appearing socially responsible to the consumer [9]. Even when their actions are not directly tied to a particular product, CSR allows them to gain or maintain a positive reputation among consumers.

CSR reflects the importance of outside stakeholders, as external pressures from NGOs and the public have had a clear influence on business actions. Some authors have also emphasized the vital role of internal stakeholders, particularly employees, in driving company actions [4], as they are in charge of creating and implementing corporate policies. Smith notes that there is an increased pressure for companies to have CSR policies [10]. Using the example of access to medicines in the pharmaceutical industry, he finds that although the motivations to engage in CSR may be normative, there is also a strategic business incentive driven by increased public pressure. He suggests that while company actions may be in the interest of public health they also “reduce the risk of eroding shareholder value” [10].

Porter and Kramer call CSR an “inescapable priority” for businesses [11]. The authors argue that CSR should not be viewed as a separate entity, which competes with the prime interests of a business (profit through its given product) but can rather be an integral component of its strategy. CSR is then viewed not as a push-back against society or a giving-in to societal interests but rather as a way to engage with society to attain the greatest benefits. CSR activities can then fall into generic activities that target areas affected by the company’s value chain or areas that are harmed as a result of the company’s activities. Furthermore, to be effective, CSR should be coordinated, measurable, and have targets just as any other business strategy.

The ATM has been evaluating select multinational pharmaceutical companies on the above criteria for the past 10 years [12]. It measures activities that would fall under the categories of value chain and mitigating harm in Porter and Kramer’s approach. Importantly, today CSR is a greater part of boardroom discussion rather than it being an isolated department of the company. The results of the ATM demonstrate that companies have begun to recognize the importance of CSR for its companies “brand” and are likely to approach it as a strategic endeavour.

### Access to medicines and CSR in Brazil

Brazil is the largest country in Latin America, with a population of around 208 million people dispersed across five regions (North, Northeast, Central-West, Southeast, South) [13]. These macro-regions are further divided into 27 states and 5,564 municipalities. In 2012, its pharmaceutical market reached almost US$26 billion after several years of double-digit growth (about 3% of the global market) [14].

The universal health care system in Brazil, Sistema Único de Saúde (SUS), was established pursuant to the 1988 Constitution which guaranteed universality, equity, and a decentralized health system [5, 15]. Throughout the 1990s, the details of decentralization were decided upon and outlined across three pieces of legislation, the Basic Operational Norms of 1991, 1993, and 1996 [16]. The responsibilities for the health system are distributed among the federal, municipal, and state governments. The Federal Government is responsible for health surveillance, management of drug registration and quality, evaluation of the health system, and management of specific health areas such as indigenous health. Municipal governments implement health actions for their citizens with funding drawn from all levels of government. In turn, the state level provides technical support and funding for municipal governments [17]. With a highly decentralized health system that includes municipalities, states, and the Federal Government, health policy-making in Brazil is a complex enterprise.

The commitment to universal access to health care is widely recognized as a defining feature of Brazil’s 1988 Constitution [5]. However, the promise of access to medicines came in the early 1990s as a result of activism for the HIV/AIDS epidemic [18]. There have been extensive governance challenges for putting the access to medicines commitment into practice. Effectiveness, efficiency, equity, and inclusiveness are all key principles, which define good governance. Extensive research on the status of access to medicines in Brazil has shown that all of these principles have been challenging to achieve. Entrenched inequalities within and between states have affected health care utilization [19] and have resulted in very different procurement prices, particularly affecting the purchasing capacity of smaller states [20]. Vieira and Zucchi found that municipalities with under 10,000 residents on average paid more per capita on medicines than larger states, with significant differences between regions as well [20]. After concluding that these differences could not be attributed to variations in health-seeking behaviours, the authors suggested that unequal negotiating power between states is likely responsible. There is also the impact of a parallel private system, which affects access to medicines by increasing out-of-pocket (OOP) costs. Although OOP payments are progressive, with the rich paying more than the poor, estimates by the WHO show that they still account for 47.2% of private spending, which is about 54% of total expenditure on health [21].

Availability is also a consistent challenge for the government in ensuring access. Providing medicines is a massive undertaking for a government, requiring not only sufficient funds to purchase the necessary medicines, but a pharmaceutical supply chain equipped to maintain sufficient stock in all pharmacies. Access and affordability studies by Bertoldi et al. have found weaknesses in both of these areas [22]. When public sector facilities were examined, availability of the lowest-priced generics ranged from 8.9 to 23.3% across six cities. This number improved when looking at branded generics (called “similares” in Brazil) (55.8 to 70.2%). There is thus a very clear need to identify ways to improve access to medicine in the country, involving both the public and the private sectors.

## Methods

To understand better how well aligned the pharmaceutical sector in Brazil is with the Governmental health priorities, we conducted a study that addressed the following questions:

1. What are the main focal areas that Brazil’s Federal Government is promoting to enhance access to medicine in the country? and,
2. How well are the CSR strategies of the Brazilian pharmaceutical industry aligned with the Government’s focal areas for access to medicine?

We conducted a literature review covering the years from Luiz Inacio Lula da Silva’s presidency in 2002 to 2016, by searching a number of databases, including SciElo, Scopus, Google Scholar, Factiva, Pubmed as well as government websites and press releases. Initial key terms used in the searches for academic papers were: Brazil, Health Policy, Health System, Innovation, Governance, Pharmaceutical Policy, Domestic, and Industry. Boolean operators and wild card functions were used to broaden the searches. The terms Medicine and Access were used in combination with the main terms but were too broad when used independently. The terms used were free-text and searches were of all-text and keywords. Focused searches for the following specific programs were expanded as they were identified: Product Development Partnerships, Health Industrial Complex, National Health Plans. The academic literature was used to identify the key health related policies during the time frame we focused on and guide searches for policies and laws established by the Federal Government, which were then retrieved from the Government records [23].

To identify the health priorities of the Federal Government in Brazil we reviewed the health policy literature both through a study of international peer-reviewed articles and original policy documents obtained from Government websites. Inclusion criteria covered two areas, the development of health policy in Brazil and the growth of the domestic pharmaceutical industry. Articles were included if they provided background and analysis on either or both of these topics. Specific searches on each of the health plans were conducted on government law databases. Five-year national health policies were examined to identify the Federal Government’s main health priorities, which clearly state what health priorities the Government wants to focus on in the specific time period.

In this research we did not want to define what constitutes CSR by Brazilian pharmaceutical firms but rather rely on how firms define their CSR activities. The selection of case study companies was done based on a consultation with all researchers. We used purposeful sampling of the firms, where we tried to maximize the variance between the firms chosen. Factors taken into consideration were: company size in terms of employee numbers and market impact, whether the firm was private or public, and other ownership arrangements. We decided to choose some of the largest Brazilian pharmaceutical companies (Ache Farmaceutica, EMS and Eurofarma) and used IMS Health Data on market share to identify those firms [24]. We included also companies that have more modest market share (Apsen Farmaceutica and Biolab Sanus Farmaceutica). We decided to include Fiocruz, a public organization, given its unique status as a government owned institution conducting research and manufacturing of health products. We therefore included in our study an examination of the CSR activities of the two production-oriented units of Fiocrus, Biomanguinhos and Farmanguinhos. The broad activities of the main Fiocruz organization were not included as its main role is research as a public health institution. We included both a publicly traded firm (Ache Farmaceutica) and privately owned ones, including a private family owned firm (EMS). As we wanted to focus on domestic pharmaceutical firms we excluded all subsidiaries of multinational firms in Brazil. We retrieved information on the sizes, ownership arrangements and focal products from the companies’ websites. In Table 1 we list the selected companies that we focused on in this study.

**Table 1:** Case Study Companies

Company	Ownership status	Founded	Size (employees)	Products
1. Ache Farmaceutica [39]	Publicly-traded	1961	>2000	Generics, branded generics, and innovative
2. Apsen Farmaceutica [40]	Private	1969	~700	Generics, branded generics, and early stage innovative
3. Biolab Sanus Farmaceutica [41]	Private	1997	2000	Branded generics, innovative
4. EMS [42]	Private, family-owned	1964	>4500	Generics, branded generics, innovative
5. Fiocruz; Biomanguinhos and Farmanguinhos [43, 44]	Public (government)	1900 as public health agency, pharmaceutical divisions founded in 1976		Generics for government purchase only
6. Eurofarma [45]	Private	1972	>6000	Generics, branded generics, innovative

Case studies were carried out through a comprehensive review of every company website, including annual reports. We collected information on company origins, company size, therapeutic categories, innovation strategy, and CSR activities and policies. We compared English and Portuguese language sites as often the English language sites contained less information. Translation was completed when this was the case. Typically information on the CSR activities was under special headings such as ‘Social Responsibilities’ or ‘Community Sustainability’. In some cases we gleaned information on CSR activities from the companies’ annual reports.

All CSR projects listed on company sites were extracted to a separate document, with links to the original source. These were coded and categorized by one author (NO), and then reviewed and revised by a second author (HT). Analysis of the companies included comparison of the categories of the CSR activities with national health policies to assess whether there was alignment with the Federal Government.

## Results

### Focal areas to enhance access to medicine in Brazil

To address our first research question: What are the main focal areas that Brazil’s Federal Government is promoting to enhance access to medicine in the country?, we examined the health policies of the Workers Party Presidents Luiz Inacio Lula da Silva (2002–2010) and Dilma Rousseff (2011–2016) and assessed their focus on access to medicines and innovation. Managing and improving health has been a governmental priority since the passing of Brazil’s constitution in 1988. Direct health policy is one way in which change can be made. However, strengthening the infrastructure and industry contributing to health can be equally important. For Brazil, this has meant focusing on two areas: the governance of the public health system and the strengthening of the domestic pharmaceutical industry.

#### An overview of health related prioritization

Critical changes affecting the governance of the public health system were made in 1998. The National Drug Policy was passed which included: reforms of the essential medicines list (RENAME) (requiring an essential medicine list (EML) guidelines for rational use of medicines), pharmaceutical surveillance (infrastructure, generics policies, drug registration), and pharmaceutical services (financing, decentralization of essential medicines and medicines availability) [25]. This policy focused on innovation and equity, transparency, accountability, and inclusiveness. Through this policy, the Federal Government pledged to provide free access to all medicines on RENAME for citizens using the SUS, although the government has yet to meet this goal [22]. The Generic Drugs Law was established in 1999 and provided a further opportunity to manage costs and improve access to medicines. This law established regulations defining the parameters of what constitutes a generic drug and therefore creating an opportunity for the domestic production of these products [26].

In addition to the National Drug Policy, there have been health plans outlining targets for the country every 3 years [27–29]. These plans have recognized the limitations of the health system and have sought to overcome them. Supplementing the health plans there has been the Health Pact focusing on the coordination and relations between the three levels of government (federal, state, and municipal) [30]. Another relevant plan, Brasil Maior, has targeted industrial policy with a component on strengthening the healthcare industry [31]. Table 2 lists some key recent Governmental policies that have been relevant to the health sector in the country and it includes both health and industrial policies.

**Table 2:** Key Health Related Policies in Brazil

Plan	Sector	Type	President
National Health Plan 2004–2007 [27].	Health	Three-year plan	Lula da Silva
Health Pact 2006 [30].	Health	Coordination across federal, state and municipal governments	Lula da Silva
Mais Saúde 2008–2011 [28].	Health	Three-year Plan	Lula da Silva
Brasil Maior [31].	Industrial	Innovation and Technology with relevant health component	Dilma Rousseff
National Health Plan 2012–2015 [29].	Health	Three-year plan	Dilma Rousseff

The Brazilian Federal Government consistently treats access to medicines as an important goal for the health system. In the 2004–2007 National Health Plan, the second goal specifically highlighted access to pharmaceutical services [27]. It established the Farmacia Popular program. This program established public pharmacies and partnered with private pharmacies to increase access to commonly prescribed medications. Medications are either free in public facilities, have an administrative co-payment in facilities under the control of Fiocruz, or have a 90% subsidy of the reference price in private facilities [32, 33]. In 2011 the program was expanded to have a component providing free medications for diabetes and hypertension. Some research has shown the free medication having substantially reduced the expenditures on medicines paid by poor people in Brazil and the poorest quintile of the population studied relied on free medicines for 80% of their needs [34].

The 2006 Health Pact included requirements for ensuring access to medicines in several specific areas of care, including health of the elderly and maternal mortality [30]. In the 2008–2011 Plan, access to medicines highlighted goals for ensuring quality and increasing the supply of medicines [28]. Finally, in the 2012–2015 Plan, one of the goals was to ensure access to medicines by improving care at both primary and specialized facilities, with another focused on strengthening the pharmaceutical assistance program which guarantees free provision of medicines on the RENAME list [29].

One approach the Federal Government has pursued to improve access to medicine is to strengthen the governance of the health care system to encourage more knowledge flow between the different components of the system. The primary focus has been on citizen participation, engaging the public in the improving the health care system [5]. There are also planned interventions that target improvements in management of specific programs as well as increasing transparency. In the 2004–2007 Health Plan, goals included provisions for improving coordination in primary care, ensuring that the system is being responsive to actual health needs, increasing participation in the management of the SUS for citizens and health workers, and increasing transparency and availability of health information [27]. In the Health Pact of 2006, the emphasis continued to be on citizen empowerment and increasing participation as well as improving accountability and management in the SUS [30]. In contrast, the 2008–2011 Health Plan emphasizes improving management of the SUS and ensuring that good quality medicines are being provided [28]. The 2012–2015 Health Plan, discussed further below, targets more specific areas of the health sector [29]. Here, targets include improving coordination of services for mental health and indigenous health, regulating the private health sector, strengthening labour and citizen relations and improving enforcement measures to increase efficiency in the SUS.

#### Industrial policy for access to medicines

Some industrial policies from the past decade demonstrate how the Brazilian Federal Government has supported the domestic pharmaceutical industry as a part of improving access to medicine in the country. References to industrial policy fostering better health can be found in both the health plans listed above as well as in in the Brasil Maior plan [31]. Where innovation is included in the health plans, the emphasis is on fostering the growth of domestic businesses and increasing the development and availability of new medicines and technologies. In 2003, the Government also established a Secretariat of Science, Technology and Strategic Inputs to promote innovation in the health sector which it further relied on in promoting industrialization of the domestic pharmaceutical sector [35, 36].

In the 2004–2007 Health Plan, there is a target for developing a national policy in science, technology and health, with a specific reference to supporting domestic technology [27]. Another point further describes the need to invest in health through industrial and technology policy with the aim of national autonomy in producing key materials. The 2008–2011 Health Plan continues with this standard, aiming to increase domestic production of pharmaceuticals and medical supplies [28]. Health-focused goals of the Brasil Maio*r* plan encourage the expansion of new businesses, strengthening and diversifying exports, and expanding funding for product development [31]. The 2012–2015 Health Plan further includes points on domestic research and production of pharmaceuticals and also prioritizes Brazil’s pharmaceutical industry interests on an international scale [29].

In 2008, Decree 12 established the so-called Health Industrial Complex (HIC), a system incorporating the major actors and their alignments, which reflects the connections between healthcare and economic development [37]. The HIC has also been incorporated as a pillar of the 2008–2011 Plan and Brasil Maior [38]. This is one of the clearest manifestations of the Government’s focus on strengthening domestic productive capacity. The HIC is a collaborative effort between the Ministry of Health, Ministry of Development, Industry and Foreign Trade and the Ministry of Science and Technology. Additional important participants are the Brazilian Development Bank and the Brazilian Innovation Agency, two of the main funding bodies. The law sets up working groups, committees, and other organizing bodies with the mandate to increase domestic innovation and foreign competitiveness. It further provides financial incentives for strategic areas, by using health system purchasing power, developing networks to support good quality and competitiveness in domestic products, and streamlining administrative/regulatory processes.

The discussion above therefore demonstrates the expectations the Federal Government in Brazil places on the domestic pharmaceutical industry to enhance access to medicine in the country and the different initiatives to ensure alignments within the pharmaceutical sector.

#### Priorities in the national health plan 2012–2015

Apart from emphasizing access to medicine and integration of health and industrial policies, the newest health plan lists further key areas for promoting health in the country [29]. In Table 3, the main priorities in the 2012–2015 Health Plan, as outlined in the document are listed.

**Table 3:** Priorities in Brazil’s 2012–2015 Health Plan

		Areas
1	Guarantee equitable and timely access to good quality medicines, through improvements of primary and specialized care	Health care, general
2	Improvement of emergency care services	Health care, general
3	Increase focus on maternal and child health through the Stork Program, emphasizing vulnerable regions	Maternal and child health; Vulnerable regions
4	Strengthen mental health networks, focusing on addictions to cocaine and other drugs	Mental health and addiction
5	Guarantee attention to health of the elderly and those with chronic conditions through strengthening of health promotion and prevention activities	Health promotion; Elderly populations
6	Implement a sub-system in healthcare focused on indigenous health in compliance with health practices and traditional medicines, maintaining respect for different cultures	Health care Indigenous health and traditional medicine
7	Use health promotion and surveillance to reduce population health risks	Health promotion and surveillance, general
8	Guarantee pharmaceutical assistance through SUS	Health access, general
9	Improve regulation of the supplementary health system, articulating the public/private relationship and ensuring more rational and good quality care	Regulation, general
10	Strengthening science, technology and innovation around the national agenda for economic, social and sustainable development with the aim of reducing vulnerabilities in access to health	Health policy, general
11	Contributing to appropriate training, allocation, qualification, valuing(?) and the democratization of work relations for SUS employees	Health education, general
12	Implementation of a new federal management model and instruments, centered around guaranteed access, participatory management and a focus on results, social participation and stable financing	Access and social participation, general
13	Qualification for direct enforcement instruments, generating gains in productivity and efficiency in the SUS	Health management
14	Promotion of Brazilian interests internationally in the field of health, sharing experiences of SUS with other countries in accordance with the Brazilian Foreign Policy	Health diplomacy

From Table 3, one can see that the priorities listed in the health plan focus on diverse areas. Many of them focus on health care in general or health care of specific populations such as maternal and child health, indigenous health and mental health. There are also health promotion and health education priorities. Further, some priorities are more policy oriented, health management or health diplomacy focused. The areas that the Brazilian Federal Government is pursuing do cover a wide spectrum. Brazilian pharmaceutical firms have, therefore, a wide range of possibilities in aligning their CSR activities with priorities of the Brazilian Government.

This section has demonstrated that the Brazilian Government has a broad focus on improving health of its population. Access to medicines is consistently on the Brazilian Government’s agenda and it is integrated into the health sector goals for the country as well as industrial policies of the country. The following section will examine the key CSR areas of Brazil’s pharmaceutical sector and the sector’s alignment with Federal Government’s health prioritization in the country.

### CSR policies’ alignment with governmental prioritization

To answer our second question: How well are the CSR strategies of the Brazilian pharmaceutical industry aligned with the Government’s focal areas for access to medicine? we examined CSR activities of six Brazilian pharmaceutical firms. As described above we looked at the websites of the firms and annual reports when available to examine to what extent they describe their engagement in CSR initiatives and the types of activities listed. Table 4 presents classification of the areas that Brazilian pharmaceutical firms list as their CSR activities.

**Table 4:** CSR Initiatives of Select Brazilian Firms

Area of programs	Number of programs	Firms active
Health care^a^	7	Apsen, Biolab, Eurofarma, Fiocruz
Health information^b^	8	Ache, Biolab, EMS, Eurofarma, Fiocruz
Health care infrastructure	4	Apsen, EMS, Fiocruz
Health care training	6	Apsen, Eurofarma, Fiocruz
Donation or discount of medicines	4	Ache, Eurofarma, Fiocruz
Senior support	2	EMS
Training (not health care related)	29	Ache, Apsen, Biolab, Eurofarma, EMS, Fiocruz
Donations food, blood, toys etc.	7	Ache, Eurofarma. Oswaldo Cruz, Fiocruz
Environmental initiatives	8	Ache, Eurofarma, Fiocruz
Social issues	10	Ache, Eurofarma, EMS, Fiocruz
Research	2	Apsen, Biolab
Child care support	4	Ache, EMS, Eurofarma, Fiocruz
Arts and culture	8	Apsen, Biolab, EMS, Fiocruz
Sport	2	Apsen, Fiocruz
Other^c^	10	Ache, Eurofarma, Fiocruz

^a^Examples include: Complementary dental care, support for cancer care and hearing and respiratory conservation programs

^b^Examples include: information on safe medication use for patients, lectures on health issues, such as on sexual health and pregnancy as well as on health policy

^c^Includes employee recognitions, visits to firms, housing support, support to deaf employees, meditation room, etc.

Table 4 shows that the firms have a number of programs aimed at fulfilling their CSR. Many of these are supporting health related activities. The programs supporting health care, health care infrastructure, health care training and seniors, all have a definite emphasis on improving access to medicine and medical services in the country. The programs do therefore align with Governmental prioritization on improving health care in Brazil and to the priority to guarantee equitable and timely access to good quality medicine. Senior’s health is also targeted by one company. It is also noteworthy how many programs focus on environmental issues, such as waste management programs, which reflect an emphasis on promoting sustainable development. Still the connection to specific Governmental prioritization is tenuous. There is, for instance, a good alignment with child health emphasized by the Brazilian Government but lack of focus on maternal health, mental health or indigenous health, which are all governmental priorities. A large proportion of the CSR activities are not directly health-related but rather focused on areas such as training in general, the arts or sports. These tend to be traditional areas supported by any industrial firms to demonstrate their good citizen role, rather than attempts to reinforce governmental health policies. In general, the case study analysis suggests that pharmaceutical firms are promoting their good citizen role to enhance general goodwill in their CSR programmes rather than reinforcing Governmental strategies to promote access to medicine and health services in specific areas.

## Discussion

The Brazilian Federal Government has set up clear health priorities that can serve as a direction for the pharmaceutical firms’ CSR activities. There has been consistent emphasis on areas such as primary health care, senior health and maternal and child health. Our research, however, identifies only loose connections between the companies’ CSR activities with the Federal Government’s health priorities as many of the firms’ programs are in areas other than health. Furthermore, programs that do emphasize health for the most part do not seem to select their area of focus based on governmental prioritizations. There seems to be clear interest from the firms examined in these case studies to develop robust CSR programming, with the potential to increase the focus on health. This motivation and the current lack of alignment therefore represent a lost opportunity to strengthen the HIC complex and make it more coherent.

We found in our investigation that Brazilian pharmaceutical companies can target their CSR programs towards the same areas as the Federal Government and thereby ideally strengthen the HIC complex and access to medicine in the country. Globally, multinational pharmaceutical companies have recognized the importance of access to medicines and CSR. Brazilian companies could do the same on a national scale. The Brazilian companies receive significant benefits from the Federal Government as it seeks to improve access via industrial policy. Should companies increase their focus on aligning CSR with national health goals, they could be in a more competitive position to receive further benefits from the Government’s continued industrial policies. Such reciprocity would demonstrate buy-in towards access to medicines and the impact of opening governance.

There are, however, a number of limitations to this research. Unfortunately information is lacking on the exact funding of the CSR programs of the firms. This, therefore, diminishes the potential of comparing the CSR emphasis of the firms as some may have few programs that are well funded whereas other firms may have many small programs. The value of the comparisons above is therefore limited to identification of the themes of the CSR programming. Further, this paper only looks at the CSR activities of the Brazilian pharmaceutical firms rather than their core activities. It is possible that there are better alignments between their core activities and governmental prioritization. By looking at the alignment through the CSR lens, it appears, however, that the activities in general lack focus and present a lost opportunity to reinforce governmental prioritisation on access to medicine. There is, thus, scope to do further research to get a fuller picture of the relationship between CSR activities of Brazilian pharmaceutical firms and the Federal Government’s health priorities.

## Conclusion

Our research article examines if and how the Federal Government of Brazil’s policy to increase access to medicine, by establishing a universal health care system and by promoting domestic pharmaceutical industrialization, may have created incentives for pharmaceutical companies to pursue a strategic CSR agenda that is in line with the Government’s objectives. We sought to determine whether such strategic approaches are currently in place by comparing Brazil’s health and industrial policies with the CSR programs of major pharmaceutical firm based in Brazil. As Brazil is undergoing significant political and economic upheavals, there is an opportunity now to observe the Brazilian pharmaceutical industry as it pursues expansion into innovative work at the same time as the Government seeks to ensure the sustainability of its health goals.

Brazil’s national health plans and industrial policies demonstrated that the Federal Government has followed a number of strategies for improving access to medicines, including strengthening health care infrastructure, developing subsidy programs, increasing transparency, and supporting product development partnerships. Case studies of six pharmaceutical firms were presented, representing both public and private companies of varying size. These suggest there are some connections between Governmental priorities and pharmaceutical firm CSR, though it is not clear that these were strategically established. There remains a significant opportunity for greater alignment, which could improve access to medicines in the country and foster a stronger relationship between the Government and industry. It remains to be seen whether Brazilian pharmaceutical companies will deepen their CSR policies to better reflect the goals of Government. This paper suggests that if they were to do so, there would be significant benefits for the Brazilian population.

## Abbreviations

ATM: Access to Medicine Index
CSR: Corporate Social Responsibility
EML: Essential Medicine List
HIC: Health Industrial Complex
MDIC: Ministry of Development, Industry and Foreign Trade
MOH: Ministry of Health
NGO: Non-Governmental Organization
OOP: Out-of-Pocket Costs
R&D: Research and Development
RENAME: Brazil’s list of Essential Medicines
